# Enhancement of X-ray-Excited Red Luminescence of Chromium-Doped Zinc Gallate via Ultrasmall Silicon Carbide Nanocrystals

**DOI:** 10.1021/acs.chemmater.0c04671

**Published:** 2021-03-18

**Authors:** Dávid Beke, Marco V. Nardi, Gábor Bortel, Melanie Timpel, Zsolt Czigány, Luca Pasquali, Andrea Chiappini, Giorgio Bais, Mátyás Rudolf, Dóra Zalka, Franca Bigi, Francesca Rossi, László Bencs, Aron Pekker, Bence G. Márkus, Giancarlo Salviati, Stephen E. Saddow, Katalin Kamarás, Ferenc Simon, Adam Gali

**Affiliations:** † Wigner Research Centre for Physics, Institute for Solid State Physics and Optics, P.O. Box 49, Budapest H-1525, Hungary; ‡ Department of Atomic Physics, Budapest University of Technology and Economics, Budafoki út 8., Budapest H-1111, Hungary; § IMEM-CNR, Institute of Materials for Electronic and Magnetism, Trento Unit C/o Fondazione Bruno Kessler, Via Alla Cascata 56/C, Povo 38123, Trento, Italy; ∥ Centre for Energy Research, Institute for Technical Physics and Materials Science, Konkoly-Thege M. út 29-33., Budapest H-1121, Hungary; ⊥ IOM-CNR Institute, Area Science Park, SS 14 Km, 163.5, Basovizza, Trieste 34149, Italy; # Engineering Department, University of Modena e Reggio Emilia, “E. Ferrari”, Via Vivarelli 10, Modena 41125, Italy; ¶ Department of Physics, University of Johannesburg, P.O. Box 524, Auckland Park, Johannesburg 2006, South Africa; ∇ CNR-IFN, CSMFO Lab, & FBK Photonics Unit, Via Alla Cascata 56/C, Povo, Trento 38123, Italy; ○ Elettra - Sincrotrone Trieste, S.C.p.A., Area Science Park, Basovizza, SS 14 Km 163.5, Trieste 34149, Italy; ⧫ Dipartimento di Scienze Chimiche, Della Vita e Della Sostenibilità Ambientale, Università di Parma, Parma 43124, Italy; †† IMEM Parma-CNR, Parma 43124, Italy; ‡‡ Department of Physics, Budapest University of Technology and Economics and MTA-BME Lendület Spintronics Research Group (PROSPIN), Budafoki út 8., Budapest H-1111, Hungary; §§ Department of Electrical Engineering, University of South Florida, 4202 East Fowler Avenue, Tampa 33620, Florida, United States

## Abstract

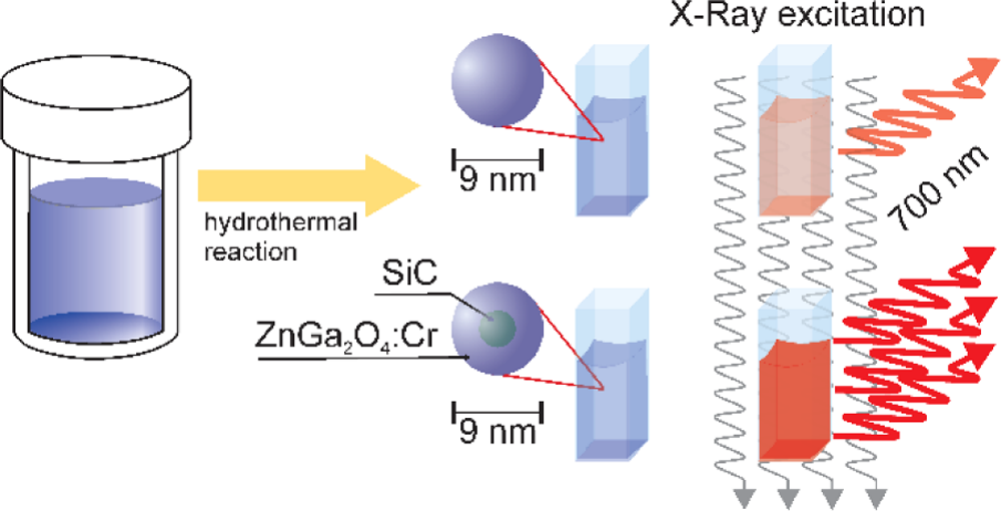

X-ray-activated near-infrared luminescent nanoparticles are considered as new alternative optical probes due to being free of autofluorescence, while both their excitation and emission possess a high penetration efficacy *in vivo*. Herein, we report silicon carbide quantum dot sensitization of trivalent chromium-doped zinc gallate nanoparticles with enhanced near-infrared emission upon X-ray and UV–vis light excitation. We have found that a ZnGa_2_O_4_ shell is formed around the SiC nanoparticles during seeded hydrothermal growth, and SiC increases the emission efficiency up to 1 order of magnitude due to band alignment that channels the excited electrons to the chromium ion.

## 1 Introduction

Zinc gallate (ZnGa_2_O_4_ or ZGO) has attracted increased attention for a broad range of optical applications owing to its excellent thermal and chemical stability and a wide band gap (≈4.4–5.2 eV).^1−3^ It is known to act as a phosphor host with one example being chromium doping of the ZGO lattice (henceforth denoted ZGO/Cr). ZGO/Cr displays a red–infrared luminescence at around 700 nm under a wide range of excitations^4,5^ when the Cr^3+^ ions replace Ga^3+^ in the crystal lattice. As a consequence, ZGO/Cr is one of the leading candidates for realizing optical imaging of vascularization, tumors, and grafted cells.^6,7^

The emission spectrum of the Cr^3+^ ion is closely related to its specific atomic structure, exhibiting three electrons in the highest energy d-orbital (*i.e.*, 3d^3^). Inside the spinel ZGO structure (representing a C_3v_ crystal field), the degenerate 3d orbital of Cr^3+^ splits into three sublevels (2e_g_ and t_2g_). The 3d^3^ multiplet states are ^4^A_2_, ^2^E, ^4^T_2_, ^4^T_1_, and ^4^T_1_ (3P). The main radiative transition occurs between the ^2^E–^4^A_2_ states and is responsible for the near-infrared emission. An unperturbed Cr^3+^ ion has two sharp photoluminescence (PL) lines without participation of vibrations called zero phonon lines (ZPLs), namely, R1 and R2, at 688.0 and 688.8 nm at room temperature (RT), respectively. Due to trigonal distortion in the ZGO crystal, the Cr^3+^ ion exhibits additional ZPLs, often marked as N1, N2, and N3 lines.^4^ The N3 line is located at around 700 nm and is attributed to Cr^3+^–Cr^3+^ pairs.^8,9^ Very close to N3, another peak can emerge, labeled n7, which originates from more complex Cr clusters. The N2 line is at 695 nm and is unambiguously connected to a first neighbor cationic anti-site defect and an inverse spinel structure around the Cr^3+^ ion.^8,10^ The origin of N1, located at 690 nm, is more controversial.^11,12^ Such defects due to trigonal distortion alter the optical properties by shifting the peak maxima and affect the emission intensity and the exciton-relaxation time.

The multiple excited states of Cr^3+^ allow exciton relaxation from the conduction band (CB) of the host material (*i.e.*, ZGO) to the lowest excited state of Cr^3+^. The perturbed local environment of the dopant can create trap states, that is, can store electrons and channel them to the dopant excited state at a later stage, creating a strong and long-lasting luminescence. This mechanism, together with the high X-ray absorption cross-section of the host, allows for high-energy excitation and makes the material suitable as a phosphor and scintillator material. Indeed, optical imaging could be advantageously carried out by using a phosphor as a luminescent probe, emitting in the red–infrared part of the spectrum upon X-ray excitation. In fact, in some cases, emission without excitation has been reported in the literature.^13^ A red–infrared emission is necessary for luminescence to pass through human tissues.^14^ By using persistent luminescent nanoparticles (NPs) and/or the capability of X-ray excitation to penetrate the body, the autofluorescence of tissue can be avoided.^3,13,15^ The use of such luminescent systems for *in vivo* imaging is of great interest to investigate pathologies in animal models and visualize deep-tissue cancer cells.

A couple of attempts were carried out to improve the emission intensity of ZGO, either by varying the Zn ratio,^5^ using Bi^3+^ doping,^16^ or mixing in conducting oxides such as In_2_O_3_.^17^ However, such methods either require precise stoichiometric control of multiple components, as the desired luminescence is very sensitive to dopant concentrations, or multiphase systems, respectively.

The most accepted method to synthesize ZGO/ZGO/Cr NPs (NPs) is the transformation of the oxides or hydroxides of the elements into crystalline ZGO. This can be accomplished by annealing an oxide or hydroxide mixture at high temperature or by applying solvothermal methods at a moderate temperature.^18−21^ The benefit of solvothermal, in particular hydrothermal, synthesis over the solid–state reaction is the significantly lower reaction temperature and a more controllable particle size.^21^ The latter is of importance for *in vivo* applications as the particle size has a huge impact on the cellular uptake, circulation time, and toxicity. However, the reaction mechanism of the hydrothermal synthesis of ZGO/ZGO/Cr has not been fully understood to date.

Here, we report on a considerable improvement of the synthesis method and optical properties of the ZGO/Cr system using silicon carbide (SiC) ultrasmall NPs^22,23^ as both seeds and optical sensitizers. The emission intensity of ZGO/Cr with a SiC core (*i.e.*, ZGO/Cr–SiC) is found to be an order of magnitude higher than that of ZGO/Cr under X-ray and UV–vis light excitation with a wavelength of 250 nm, whereas it is 2 times higher under 290 nm UV–vis light excitation. Such an enhancement is significantly larger than that observed in previous reports^16,24−26^ while, to the best of our knowledge, excitation wavelength-dependent enhancement has not been reported to date. Reaction kinetic studies suggest that ultrasmall SiC NPs can reduce the formation energy barrier of ZGO/Cr during hydrothermal synthesis allowing for faster ZGO/Cr particle growth at the early reaction stage and channels the excitons to the Cr^3+^ ions in the lattice, thus improving the emission intensity significantly under high-energy excitation. Such a seeding effect is found to be size-selective, and only ultrasmall SiC NPs with a diameter below 3 nm were found to participate in the colloid reaction.

## 2 Results

### 2.1 General Physical Properties of NPs

**Figure 1:**
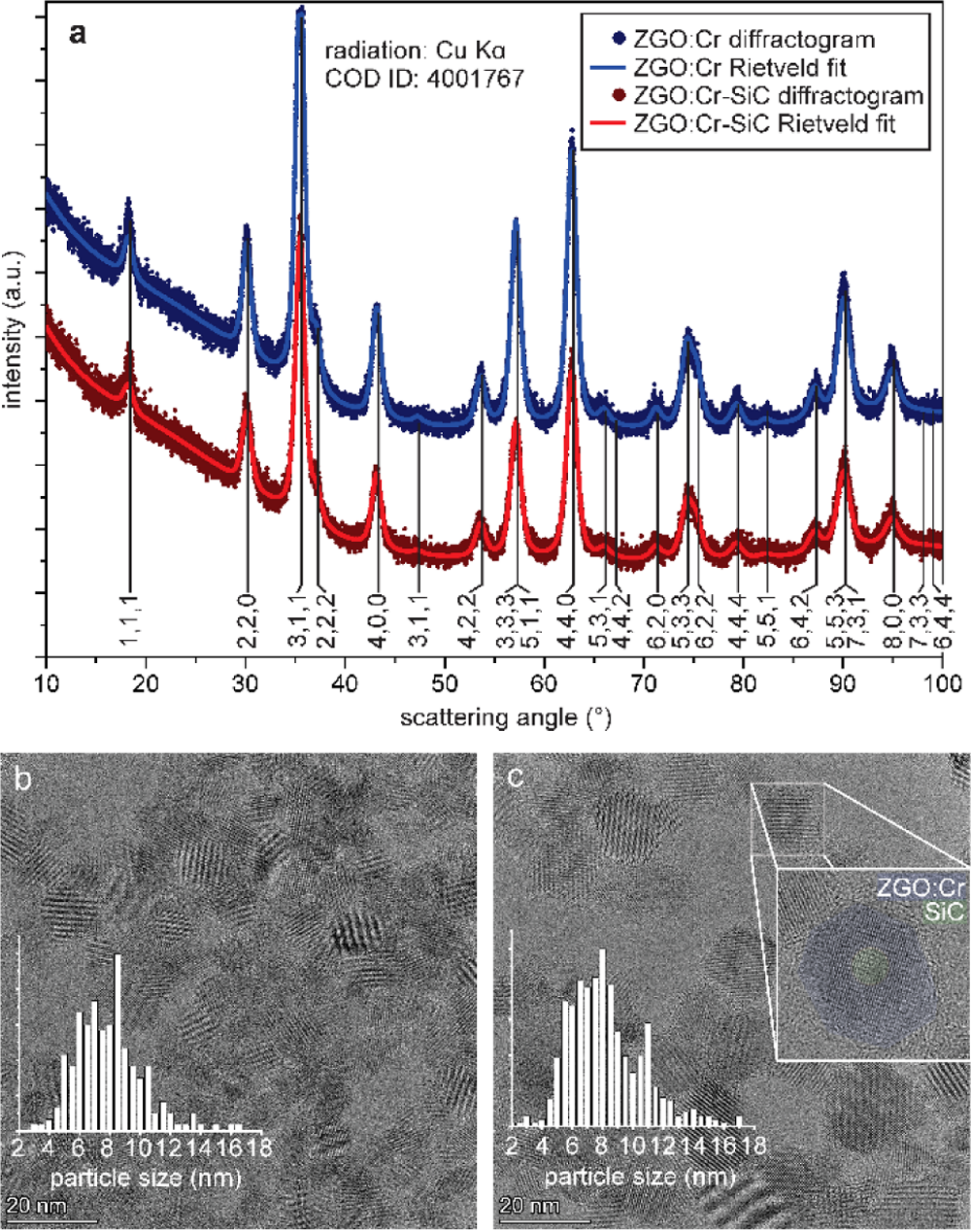
(a) XRD of ZGO/Cr and ZGO/Cr–SiC NPs with reference to the ZnGa_2_O_4_ crystal structure (COD). (b) HR-TEM images of ZGO/Cr and (c) ZGO/Cr–SiC NPs. The insets in (b) and (c) show the size distribution calculated from several HR-TEM images.

The comparison of the structure, crystallinity, size, and morphology of ZGO/Cr and ZGO/Cr–SiC results in many similar features (Figure 1). The broad reflections of the X-ray diffraction (XRD) pattern in both cases (Figure 1a) correspond to the cubic spinel crystal structure of ZnGa_2_O_4_ [Crystallography Open Database (COD) ID 4001767, space group , *a* = 8.35 Å]. The Rietveld analysis of the profile indicated 10 and 9.5 nm crystallite size for the ZGO/Cr and ZGO/Cr–SiC samples, respectively. The Rietveld refinement of the properly restricted tetrahedral and octahedral site occupancies for Zn^2+^, Ga^3+^, and Cr^3+^ yielded a ∼10% degree of inversion of the spinel structure (continuous parametrized transition from the spinel to the inverse spinel structure). Reflections from the SiC seeds, which should appear as 3 times more broadened reflections with respect to ZGO, are not visible in the pattern due to the low volume fraction of SiC (2.7%), evidencing that SiC seeding does not perturb the XRD pattern of ZGO/Cr. The lattice spacing, measured *via* high-resolution transmission electron microscopy (HR-TEM) analysis (Figure 1b,c) and selected area electron diffraction patterns (EDS) patterns (not shown), also confirms that the ZnGa_2_O_4_ structure was synthesized.

Scanning electron microscopy (SEM), EDS, and TEM–EDS confirm the presence of Zn, Ga, O, Si, and C in the ZGO/Cr–SiC NPs and Zn, Ga, and O in the ZGO/Cr NPs (Table 1). The size-distribution measurements from HR-TEM analysis gave very similar results to XRD, namely, 9 nm for ZGO/Cr and 9.5 nm for ZGO/Cr–SiC (insets, as shown in Figure 1b,c). The hydrodynamic sizes measured by means of dynamic light scattering (DLS) in aqueous solution were 28 and 29 nm with a dispersity of 3.2 and 3.4 for ZGO/Cr and ZGO/Cr–SiC NPs, respectively. The significant difference between the sizes measured by XRD/HR-TEM and DLS can be explained by either cluster formation/aggregation as a result of the enthalpy minimization by decreasing the surface free energy. This leads to the presence of large agglomerates which hamper the detection of smaller individual particles by DLS^19^ or interparticle interaction that reduces the average diffusion speed of the particles causing an overestimation in the particle size.^27^ Nevertheless, the DLS data correlate well with the other parameters measured in reaction kinetics experiments implying reasonable accuracy.

**Table 1:** Results of Electron Diffraction Spectroscopy (EDS) and Atomic Absorption Spectroscopy (AAS) Elemental Analysis of ZGO/Cr and ZGO/Cr–SiC NPs

	at. % (SEM–EDS)		at. % (TEM–EDS)	
element	ZGO/Cr	ZGO/Cr–SiC	ZGO/Cr	ZGO/Cr–SiC
Zn	9.7	6.5	21.6	9.9
Ga	16.7	14.3	24.2	19.0
O	60.3	43	53.4	70.2
Si	10.5	15.1	0.6	0.8
C		21.3		
Cr			0.09	0.08

	AAS concentration (μg/mg)	
	ZGO/Cr	ZGO:Cr–SiC
Cr (×10^–6^ mol/L)	0.189	0.195
Cr (×10^–7^ mol/L)	0.001	0.100

The HR-TEM image, as shown in Figure 1c, reveals the core–shell structure of ZGO/Cr–SiC NPs since Si and C are lighter elements than Zn and Ga, causing a brighter NP center in the TEM images. There was no sign of free SiC NPs and/or SiC NPs aggregated onto the surface of the ZGO NPs. Based on the HR-TEM images, we have calculated that 80% of the particles have core–shell structures. Additional TEM images and extended discussion can be found in the Supporting Information.

**Figure 2:**
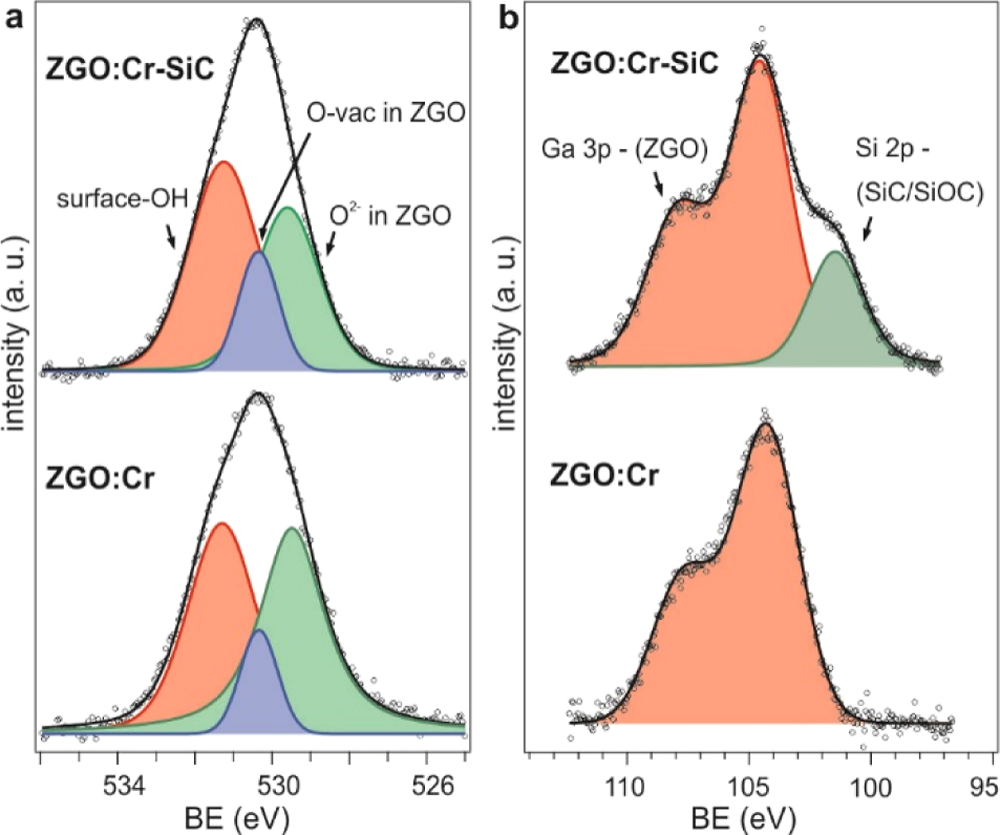
Fitted XPS (a) O 1s and (b) Ga 3p–Si 2p core level spectra of ZGO/Cr and ZGO/Cr–SiC NPs.

X-ray photoemission spectroscopy (XPS) chemical analysis of ZGO/Cr and ZGO/Cr–SiC is reported in Figure 2, where fitted O 1s and Ga 3p + Si 2p core level spectra are shown. The spectral features of the O 1s peak, as shown in Figure 2a, are similar for both samples and correspond to O^2–^ species (green component) and oxygen vacancies (O-vac; blue component) of ZGO located at binding energies (BEs) of 529.6 and 530.3 eV, respectively, and surface hydroxide groups (red component) at BE = 531.3 eV. As shown in Figure 2b, the Ga 3p peak (red component) is observed at 104.3 eV (104.5 eV) for ZGO/Cr (ZGO/Cr–SiC). Different to ZGO/Cr, the ZGO/Cr–SiC sample exhibited an additional peak (green component) at 101.4 eV, which is attributed to the Si 2p core level of SiC/SiOC.

Despite the similar crystal structure and particle geometry, ZGO/Cr–SiC NPs exhibit unambiguously brighter luminescence upon the same conditions (see results below). In order to understand such differences, we studied the local environment of the Cr^3+^ ion, as well as the reaction kinetics, along with the excitation and relaxation paths in both systems.

### 2.2 Optical Properties

**Figure 3:**
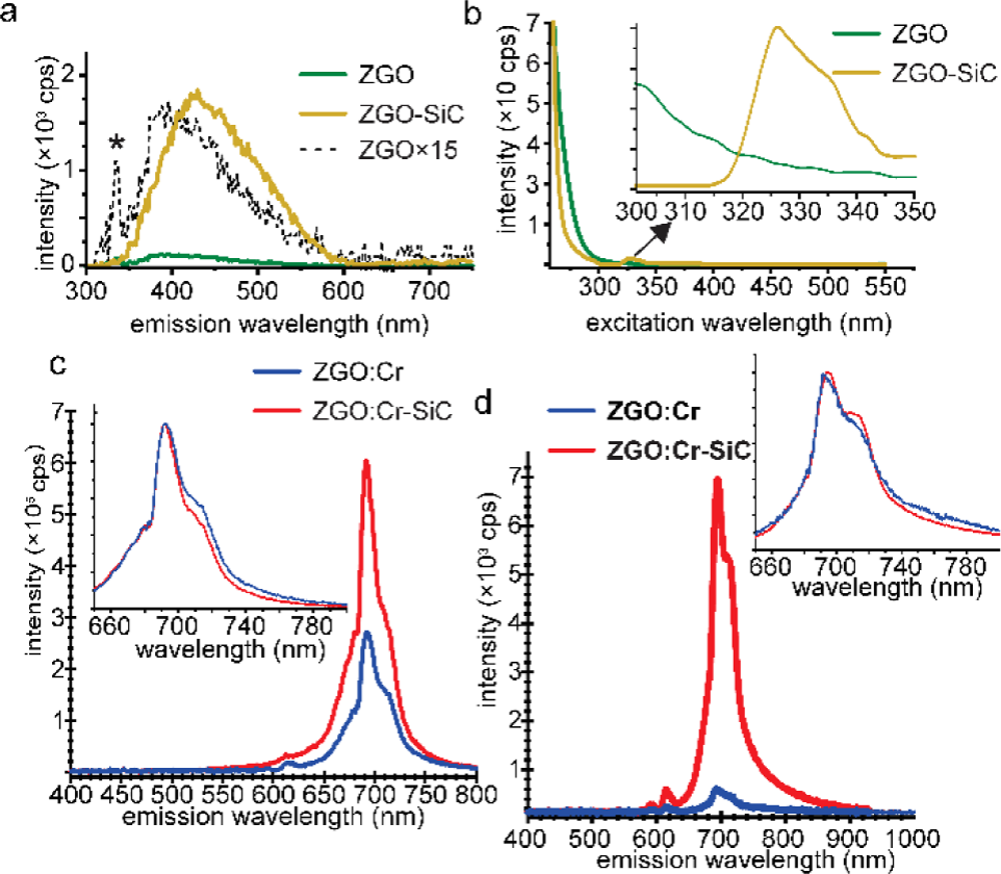
(a) RT-PL (290 nm UV illumination) and (b) PLE spectra of ZGO and ZGO–SiC NPs without Cr^3+^ ion doping. The asterisk in (a) marks the Raman peak of water, whereas the black curve in (a) represents the ZGO spectrum multiplied by 15 to compare luminescence shapes and maxima. The inset in (b) shows the PLE peak of ZGO–SiC originating from the SiC NPs. (c) RT-PL (290 nm UV illumination) and (d) XEOL spectra (non-monochromatized X-rays, 5–30 keV) of ZGO/Cr and ZGO/Cr–SiC NPs. The inset in (b) shows the PLE peak of ZGO–SiC originating from the SiC NPs. Insets (c,d) show spectra normalized to peak intensity.

The optical properties of ZGO and ZGO–SiC NPs were carefully studied with and without Cr^3+^ ion doping. Without Cr^3+^ ions, the bare ZGO NPs exhibit a weak broad PL peak centered at around 400 nm (see Figure 3a), whereas ZGO–SiC NPs show enhanced emission properties with an emission maximum at 435 nm. It is known that ZGO usually exhibits a blue–green emission, due to a self-activation center,^28^ and SiC NPs yield luminescence at 435 nm when carboxylic surface groups are coordinated with alkali-metal ions.^29^ Even though the two aforementioned PL spectra overlap, the PL excitation (PLE) spectrum of ZGO–SiC NPs shows an additional excitation peak at around 320 nm (Figure 3b), where SiC NPs have a maximum, suggesting a SiC-sensitizing effect in a Cr-free ZGO structure. However, these broad emission lines disappear when Cr is present during the reaction (see Figure 3c,d).

In the presence of Cr^3+^, both room-temperature PL (RT-PL; upon 290 nm UV illumination, see Figure 3c) and XEOL spectra (upon non-monochromatized X-ray radiation, see Figure 3d) show the typical emission by Cr^3+^ ions in a ZGO structure at a wavelength around 700 nm, with the absence of other emissions for ZGO/Cr and ZGO/Cr–SiC NPs. In addition to the main ZPLs, Stokes and anti-Stokes phonon sidebands (PSBs) appear as low- and high-energy shoulders in the PL spectra, broadening the entire emission spectra.

Under 290 nm UV illumination (Figure 3c), ZGO/Cr–SiC NPs show a two-fold higher PL emission intensity than ZGO/Cr NPs, in both colloid solution and powder form. Interestingly, the XEOL emission intensity of ZGO/Cr–SiC NPs under hard X-ray radiation (*i.e.*, non-monochromatized 5–30 keV X-ray light in Figure 3d and monochromatized high-flux 21 keV X-ray light in the Supporting Information) and 250 nm UV illumination (see the Supporting Information) is more than an order of magnitude higher than the emission from ZGO/Cr NPs. Such an enhancement is significantly larger than previously reported,^16,24−26^ while, to the best of our knowledge, excitation wavelength-dependent enhancement has not been reported to date.

### 2.3 Local Environment of Trivalent Chromium

**Figure 4:**
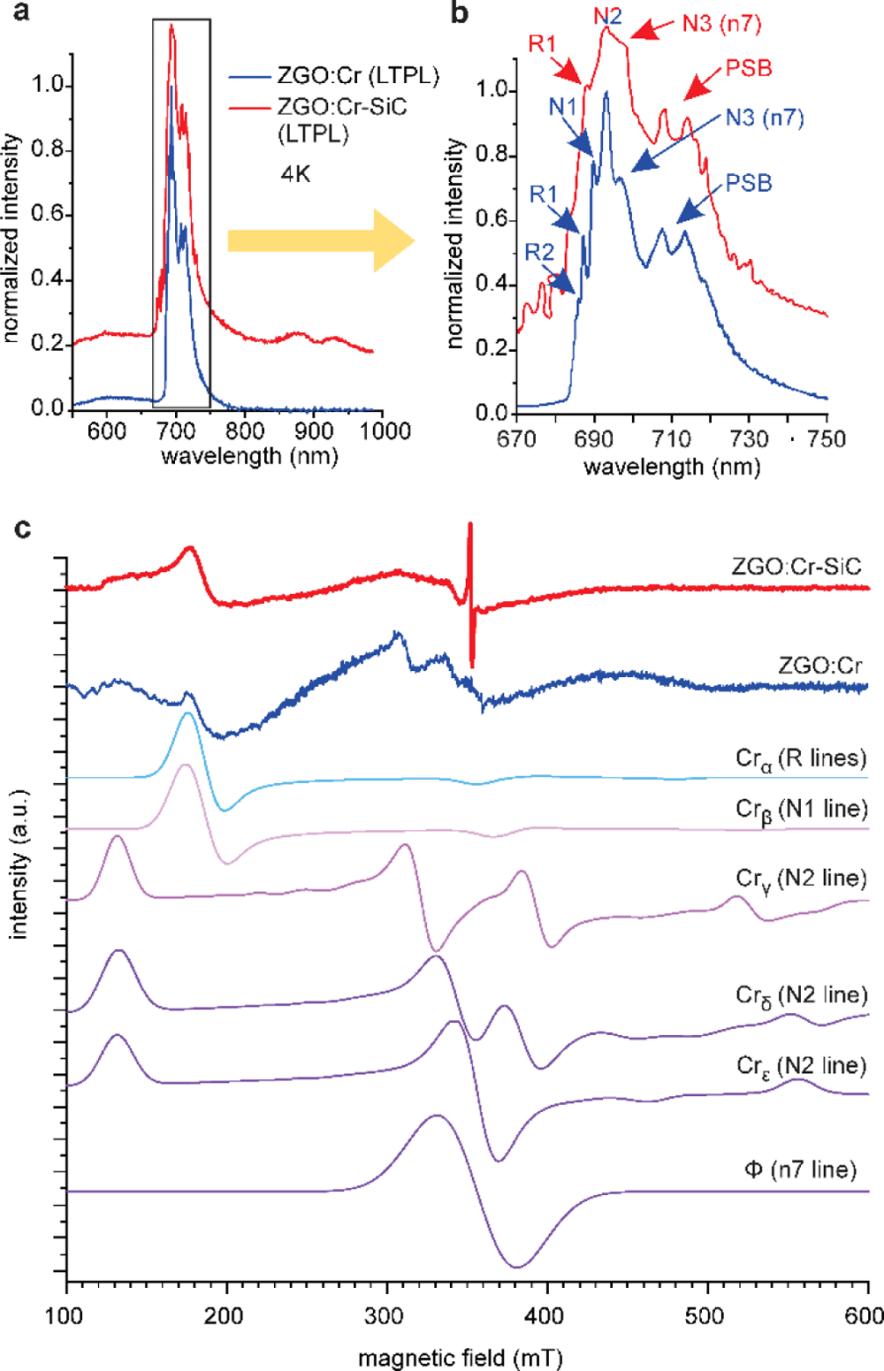
(a) LT-PL spectra of ZGO/Cr and ZGO/Cr–SiC NPs. (b) LT-PL around 700 nm, showing the Cr peak with the corresponding ZPLs. (c) ESR spectra of ZGO/Cr and ZGO/Cr–SiC NPs, and the components for fitting from ref (4). The corresponding PL lines are indicated in parentheses.

It is commonly known that the local environment of an emission center alters its optical properties. The absence of the SiC emission peak in ZGO/Cr–SiC NPs implies a strong interaction between the NPs’ SiC core and Cr^3+^ ions. Therefore, the different local environment around Cr^3+^ appears in ZGO/Cr and ZGO/Cr–SiC NPs. The fine structure of the observed PL, as well as electron spin resonance (ESR) spectroscopy, can probe the local environment.^4,8,12,13,30^ Under UV illumination at RT (Figure 3c), the only difference between ZGO/Cr and ZGO/Cr–SiC is a small increase in the peak broadening due to PSBs (Stokes) in ZGO/Cr NPs. Under X-ray excitation (Figure 3d), the emission maximum is slightly red-shifted. The shift is more intense in ZGO/Cr–SiC NPs although the difference between the samples is as small as 2 nm. Nevertheless, ZGO/Cr and ZGO/Cr–SiC NPs show similar transitions. At a low temperature of 4 K [see the low-temperature PL (LT-PL) spectra in Figure 4a,b], the ZPLs (*i.e.*, R1, R2, N1–N3, and n7) are mostly resolved, due to decreased thermal broadening. At this temperature, the N2 line has the highest intensity in either sample. By comparing the LT-PL spectra of the two samples, it can be seen that the N1 line is visible in ZGO/Cr but not resolved in ZGO/Cr–SiC. Furthermore, the N3 (and/or n7) line (corresponding to Cr^3+^ cluster defects) has a significantly higher contribution in ZGO/Cr–SiC NPs than that in ZGO/Cr NPs, and the whole spectrum is broader due to the higher contribution of the PSBs. In addition to the sharp PL lines, the ZGO/Cr–SiC sample exhibits two unknown weak, but broad, peaks at 880 and 935 nm.

The ESR measurements (Figure 4c) were used to correlate the centers, as found in the LT-PL spectra (Figure 4a,b). Gourier *et al.*(4) extensively studied the origin of the red luminescence of Cr^3+^ ions in ZGO using mostly Q-band ESR spectroscopy. They identified six types of Cr^3+^ ions exhibiting different neighboring defects. We use the reported parameter set as a starting point to simulate our X-band ESR spectra of the ZGO/Cr and ZGO/Cr–SiC NPs. Similar to the LT-PL spectra, the ESR spectra of ZGO/Cr are found to be more complex than ZGO/Cr–SiC and need to be reconstructed using five different parameters for each Cr center. In contrast, the ZGO/Cr–SiC spectra can be reconstructed with three parameters, with a high contribution from the so-called Cr_γ_ and φ centers. Gourier *et al.*(4) associated the Cr_γ_ ESR center with the PL signal labeled as the N2 line and the φ ESR center with the n7 line.

Vibrational spectroscopy such as Raman and FTIR can reveal local differences in the crystal structure and was used as a probe of the local environment of the Cr^3+^ ions from the crystal side. Our Raman and FTIR data show higher inverse spinel concentration in ZGO/Cr–SiC than that in ZGO/Cr (see the Supporting Information). Due to the fact that the inverse spinel crystal structure is related to the N2 line, the results of Raman and FTIR confirm the increased number of N2-type defects in ZGO/Cr–SiC.

### 2.4 Investigation of Hydrothermal Synthesis

#### 2.4.1 Variation of Reaction Parameters

**Figure 5:**
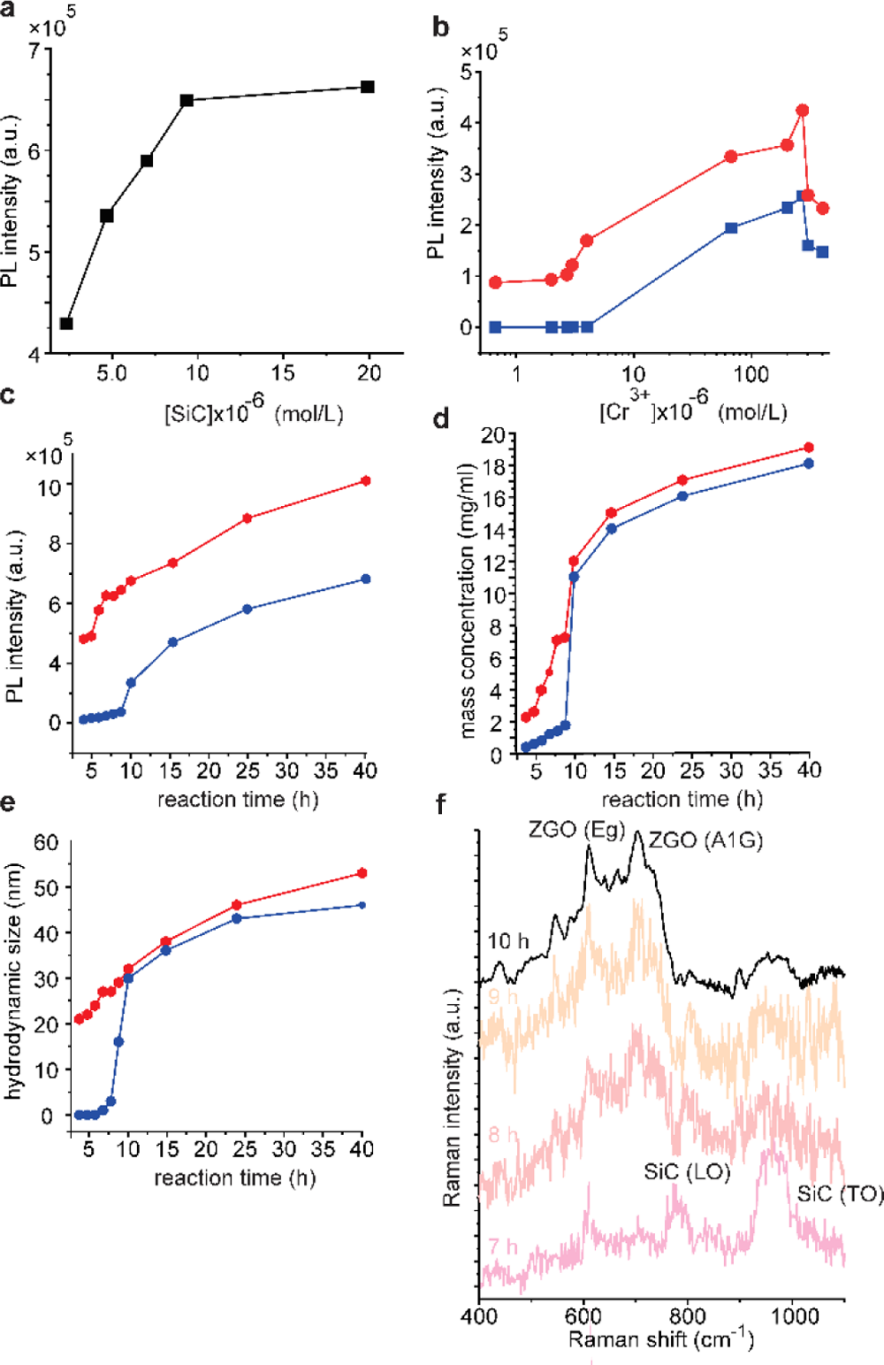
(a) RT-PL intensity *vs* nominal SiC concentration for ZGO/Cr–SiC NPs and (b) RT-PL *vs* nominal Cr^3+^ concentration for ZGO/Cr (blue) and ZGO/Cr–SiC NPs (red). The growth kinetics were studied as a function of (c) emission intensity, (d) mass concentration, and (e) hydrodynamic size. (f) Raman spectra of ZGO/Cr–SiC NPs after different reaction times. The ZGO grown on SiC crystallizes only after 10 h similar to the unseeded sample.

The reaction parameters of the hydrothermal synthesis were evaluated *via* the near-infrared emission originating from Cr^3+^ ions, as seen in the PL spectra (integrated between 680–720 nm) in Figure 5a–c. It is noteworthy that a reaction temperature of at least 200 °C was needed for detectable luminescence at 10 h of reaction, whereas the pH had no significant effect on the product in the pH range of 6–10. More specifically, the same amount of precipitate was formed after 10 h for each pH value, even for acidic pH, without hydroxide precipitation.

The emission intensity of Cr^3+^ PL *versus* nominal SiC and Cr^3+^ concentration can be seen in Figure 5a,b, respectively. The PL intensity gradually increases with increasing SiC concentrations (Figure 5a) in the reaction mixture and reaches a plateau at around 1 × 10^–5^ mol/L [SiC]. The effect of [Cr^3+^] displayed the same trend for ZGO/Cr and ZGO/Cr–SiC around the optimal Cr^3+^ concentration (blue and red curves, as shown in Figure 5b, respectively). It should be noted that ZGO/Cr–SiC NPs show luminescence even when the Cr^3+^ concentration decreased by an order of magnitude, whereas luminescence of ZGO/Cr NPs was only detected above a nominal Cr^3+^ concentration of 1 × 10^–5^ mol/L. The Cr^3+^ content in the NPs was determined by means of high-resolution AAS for the two nominal concentrations 1 × 10^–6^ and 1 × 10^–7^ mol/L. For 1 × 10^–6^ mol/L [Cr^3+^], AAS indicated marginal differences between the samples with and without SiC (see Table 1). However, when the nominal Cr^3+^ concentration was reduced to 1 × 10^–7^ mol/L, the ZGO/Cr–SiC NPs displayed a much higher Cr^3+^ concentration than that for the ZGO/Cr NPs. The very low Cr concentration (as measured by AAS) for ZGO/Cr agrees well with the undetectable PLfor low (nominal) Cr concentrations (blue curve, as shown in Figure 3b), while AAS and PL analyses indicate that SiC promotes Cr^3+^ ions to be built into the ZGO crystal.

#### 2.4.2 Studies on Reaction Kinetics

Following PL intensity evolution through the reaction time (Figure 5c), the plot shows a sigmoidal colloid formation kinetics for ZGO/Cr NPs (blue curve, as shown in Figure 5c). For ZGO/Cr–SiC NPs (red curve, as shown in Figure 5c), the exponential increase begins after a much shorter reaction time (6 h), and the overall graph shows a more complex shape with a double sigmoidal characteristic. We found a very similar trend for mass concentration (Figure 5d) and hydrodynamic size (Figure 5e). Indeed, the Lifshitz–Slyozov–Wagner (LSW) model^31,32^ describes the growth of individual colloidal particles, assuming that the enlargement rate (*i.e.*, growth kinetics) of colloidal NPs is determined by two mechanisms including the initial diffusion of precursor species from the bulk solution to the surface of the growing NP and the following reaction of the precursor species on the surface of the NP. The colloid particle formation and growth kinetics are described by a reaction-limited particle formation at the early stage, followed by fast particle growth due to the autocatalytic surface reaction and a diffusion-limited particle growth^33^ later on. According to Wu *et al.*,^34^ the diffusion constant can be extracted from the end of the reaction. We obtain the same slope for the diffusion coefficient determination showing no differences at the very late (diffusion-limited) stage between ZGO/Cr and ZGO/Cr–SiC NPs (see the Supporting Information), which is expected when SiC NPs are already covered, and the oxide grows on the same oxide surface.

Even though SiC NPs can catalyze the oxide formation, ZGO/Cr and ZGO/Cr–SiC have the same particle size after 10 h of reaction with similar crystallinity. Following the reaction with Raman spectroscopy (Figure 5f), it can be seen that the oxide in ZGO/Cr–SiC NPs is mainly amorphous at the early stages. Furthermore, the variation of particle size between 7 and 10 h of reaction is only 18%, and the sample crystallinity becomes much more pronounced after 10 h of reaction.

Larger SiC NPs (*i.e.*, ø = 4–6 nm) do not participate in core–shell structure formation and do not enhance the luminescence, and the particle growth follows the same single sigmoidal kinetics as that of the SiC-free sample (see the Supporting Information), indicating that the size, or the size-selective properties^23,35^ of the seeds, has considerable impact on the reaction.

### 2.5 Energy Levels in ZGO/Cr–SiC

The local environment of Cr^3+^ ions shows small differences when SiC is present in the host NPs (see Figure 4). Indeed, a different local environment of Cr^3+^ ions in ZGO/Cr compared to ZGO/Cr–SiC is a possible explanation for the different PL intensities and the resulting luminescence enhancement in ZGO/Cr–SiC NPs. However, the n7 defect (whose concentration was found to be higher in the presence of SiC NPs) usually decreases the emission intensity^36^ and such a difference cannot explain the excitation wavelength-dependent enhancement.

**Figure 6:**
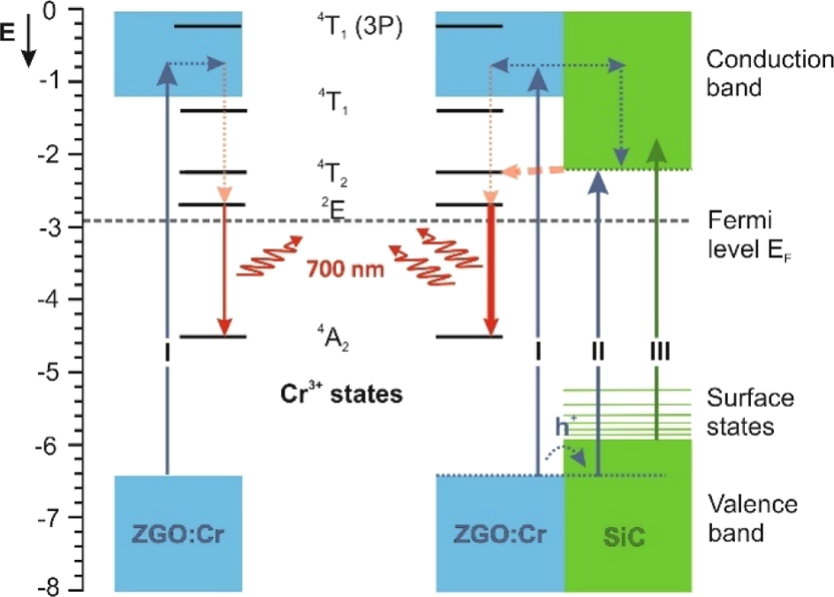
Energy levels of ZGO/Cr and ZGO/Cr–SiC NPs. The energetic positions of the valence band (VB) onset of ZGO/Cr and SiC were determined by UPS measurements, and the onset of the conduction band (CB) for ZGO/Cr was determined by its band gap value (5.2 eV, see the Supporting Information), whereas the CB onset of SiC was estimated considering its optical band gap, as measured by PLE. The energetic positions of the Cr^3+^ states were determined by PLE. Blue and green arrows indicate different excitation pathways I–III, whereas non-radiative relaxations and electron/hole transfer are marked by dashed lines.

The relative energy positions of the ground and excited states of the different constituents (*i.e.*, ZGO, Cr^3+^, and SiC) can reveal the possibility of electron transfer in the system. To visualize the relative positions of the ground and excited states, we calculated the energy levels of ZGO/Cr and ZGO/Cr–SiC from PLE and UPS measurements (see the Supporting Information for more details), as shown in Figure 6. The energy positions of the Cr^3+^ states are also displayed in Figure 6, and possible excitation and relaxation pathways between the ZGO/ZGO–SiC host and Cr^3+^ ions are indicated by arrows.

In the energy level diagram, it can be seen that the Fermi level lies just below the ^2^E excited state of Cr. The energy difference between the ^4^A_2_ ground state of Cr and the CBM_SiC_ is about 2.4 eV, whereas the energy difference between VBM_ZGO/Cr_ and CBM_SiC_ is about 4.2 eV. The energy difference of 4.2 eV is consistent with the extra peak appearing at around 320 nm in the PLE of the ZGO–SiC system and its disappearance when Cr is present (Figure 3b), suggesting electron transfer from SiC to Cr^3+^. The constructed energy diagram does not consider any distortion caused by the local environment; however, it can be seen that, upon excitation, the positions of the excited states enable electron transfer from SiC to Cr^3+^ since the difference between CBM_SiC_ and the ^4^T_2_ state of the Cr^3+^ ion is only 0.2 eV. For excitation energies larger than the band gap of ZGO (*e.g.*, X-ray and 250 nm UV illumination), the photons can also excite the ZGO host, enabling new types of relaxation pathways (*i.e.*, excitation of VBand core electrons by X-rays), while 290 nm UV illumination excites only the SiC VBand Cr^3+^ ions/defects. On the other hand, SiC and ZGO/Cr create a type-I heterojunction enabling electron and hole transfer between ZGO/Cr and SiC upon excitation with wavelengths above the band gap of ZGO. The excitation wavelength-dependent emission enhancement is supported by quantum yield (QY) measurements of ZGO/Cr–SiC NPs as a function of the excitation wavelength (see the Supporting Information), where an enhanced QY was found for wavelengths longer than 260 nm (4.8 eV), corresponding to the band gap of ZGO.^1−3^

It should be noted that the energy-level diagram can also explain the weak, broad infrared peaks found in the LT-PL spectrum of ZGO/Cr–SiC (Figure 4a) because the energy difference between VBM_SiC_ and the ^4^A_2_ ground state (about 1.4 eV) matches these infrared PL peaks. The reason for the observed doublet feature, as shown in Figure 4a, might be due to ground-state splitting.

## 3 Discussion

The reaction mechanisms of the hydrothermal synthesis of ZGO/ZGO/Cr can be better understood with the results of our detailed reaction kinetics studies. Even though ZGO synthesis is described as the hydroxide phase transforming into the corresponding oxide phase *via* an endothermic dehydration reaction,^37^ our findings of particle formation without hydroxide precipitation and correlation with the LSW model, suggest a solution phase reaction regardless of the presence of the hydroxide. Reaction-limited nucleation creates an amorphous oxide that crystallizes during the second synthesis step. The difference in growth kinetics in the presence of SiC NPs suggests seeded nucleation and growth that does not affect the overall crystallization step. However, ZGO possesses increased stability on the surface of the SiC NPs, which results in a more uniform local environment around the Cr^3+^ ions in the product.

Interestingly, seeding *via* SiC NPs had a minor effect on the final particle size and yield, which highlights the importance of the crystallization step in particle formation. The exothermic reaction probably provides the necessary energy for stable and rapid NP formation. We rule out the possibility of secondary nucleation as an explanation of the double sigmoidal kinetics of ZGO/Cr–SiC NPs because the same mean particle size and dispersity were observed by DLS and TEM.

LT-PL and ESR analyses confirmed that SiC NPs coordinated with Cr^3+^ ions and increased the concentration of Cr-cluster-type defects. On the one hand, this had a minor effect on the optical properties in the optimal Cr^3+^ concentration range as the majority of the Cr^3+^ ions are in the N2-type local distortion in either sample. On the other hand, the coordinating effect of SiC NPs resulted in PL even at low Cr^3+^ concentration. This indicates that the Cr-cluster-type defect formation by SiC NPs becomes important in PL processes for low Cr^3+^ concentration.

The core–shell structure of ZGO/Cr–SiC NPs creates a type-I heterojunction that, together with the band alignment of the Cr^3+^ excited states and the SiC CB, opens up new excitation pathways (marked by II and III in Figure 6) and channels excitons more efficiently to the Cr^3+^ excited states, with significantly increasing emission efficiency of the system. The ZGO sub-band gap excitation (*e.g.*, 290 nm UV illumination) only enables SiC–Cr interactions, which doubled the emission intensity with respect to ZGO/Cr. Excitation above the ZGO band gap (*e.g.*, 250 nm UV and hard X-ray illumination), however, enabled ZGO/Cr–SiC interactions and, due to the heterojunction structure, the emission intensity was an order of magnitude higher in the presence of SiC.

## 4 Conclusions

We found that seeded nucleation using ultrasmall (<4 nm) SiC NPs can be used for undoped (ZGO) and Cr-doped ZnGa_2_O_4_ (ZGO/Cr) NP hydrothermal growth to improve the structural and luminescence properties of the particles. The particle size of the SiC NPs has considerable influence on seeding, that is, only SiC NPs with diameters below 3 nm act as seeded nucleation sites. The present growth kinetics study shows that nucleation and crystallization are different processes, and seeding *via* ultrasmall SiC NPs propagates only the nucleation. Furthermore, SiC NPs interact with Cr^3+^ ions, which causes an increased concentration of Cr cluster-type defects in ZGO/Cr–SiC NPs.

When illuminated by X-ray light, the ZGO/Cr–SiC NPs showed remarkable enhancement of Cr^3+^ ion red luminescence (≈700 nm), namely, by an order of magnitude with respect to ZGO/Cr. A similar enhancement of red luminescence was found, when 250 nm UV illumination was used, whereas 290 nm UV illumination enhanced it only by a factor of 2. A detailed analysis of the electronic properties provides evidence that the ultrasmall SiC NPs form a type-I heterojunction ZGO/Cr–SiC nanostructure, promoting the channeling of excitons to the sensitizer (Cr^3+^). Such a sensitizing effect by ultrasmall SiC NPs explains the increased luminescence intensity. We envisage that the strong response to X-ray light makes the luminescent ZGO/Cr–SiC NPs potentially promising for *in vivo* imaging and X-ray-excited anti-cancer treatments, where high brightness is a fundamental prerequisite.

## 5 Experimental Section

### 5.1 Materials

SiC NPs with different diameters, namely, ultrasmall (ø = 1–3 nm) and larger SiC NPs (ø = 4–6 nm), were synthesized in our laboratory, and the synthesis and properties, as a function of size, can be found in our previous reports.^23,38,39^

We used nitrates, namely, Zn(NO_3_)_2_·6H_2_O (Sigma, reagent grade, 98%), Ga(NO_3_)_3_·*x*H_2_O (Sigma, trace metals basis, 99.9%), and Cr(NO_3_)_3_·9H_2_O (Sigma, trace metals basis, 99%) as cation sources for ZGO synthesis. Aqueous ammonia solution (32%, VWR, HiPerSolv, CHROMANORM) and HCl (37%, VWR, Anal-R Normapur) were used for pH adjustment and cleaning. High-purity 18 MΩ cm Millipore type 1 water (hereafter, DI water) was used for solvent preparation, dilution, and cleaning. 2-Propanol (IPA) (VWR, 99.8% HiPerSolv, CHROMANORM) was used for particle precipitation.

### 5.2 Synthesis Procedures

Undoped (ZGO) and chromium-doped ZnGa_2_O_4_ (ZGO/Cr), without and with SiC NPs (ZGO/Cr–SiC), were prepared by a hydrothermal method based on the report by Li *et al.*(19) Briefly, 1 mL of Zn(NO_3_)_2_, 1 mL of Ga(NO_3_)_3_, and 1 mL of Cr(NO_3_)_3_ solutions from 2 mol/L Zn(NO_3_)_2_, 2 mol/L Ga(NO_3_)_3_, and 4 mmol/L Cr(NO_3_)_3_ aqueous solutions, respectively, were mixed, and the total volume was adjusted to 15 mLwith DI water for ZGO/Cr or an aqueous SiC NP solution (1.5 × 10–5 mol/L) for ZGO/Cr–SiC. A 2 mL aliquot of ammonium hydroxide (32%) was added to the mixtures to achieve a pH of 9. After 30 min of stirring, the precursors were sealed into a PTFE-lined autoclave and annealed at 220 °C for 10 h. The white precipitate obtained after the reaction was centrifuged out, washed with ammonia solution, DI water, HCl–IPA (0.1 mol/L HCl and isopropyl alcohol in a 1:10 ratio), and IPA after which it was dried at 60 °C. Samples were then redispersed in DI water or pressed into a pellet for characterization. The SiC and Cr^3+^ concentrations, reaction temperature and time, and pH were varied in order to find the best synthesis conditions and to study the reaction kinetics.

### 5.3 Sample Preparation for PL and XEOL Measurements

We strove for identical sampling concentrations to accurately compare the colloid solutions of the different samples via PL, UV–vis, and XEOL measurements. To achieve that a 5 mg/mL aqueous solution was prepared by diluting the product of *ca.* 10 mg/mL. The weight concentration was measured with a Kern model 770–15 analytical balance from a 1.00 mL sample volume and an MYA 2.4Y microbalance from a 0.10 mL sample volume. Each measurement was repeated five times. Due to the smaller density of SiC compared to ZGO, the same particle size causes some increase in the concentration for ZGO/Cr–SiC. Considering spherical particles of 9.5 nm diameter with a 2 nm core diameter, this increase was 11%, which was taken into account.

## Supporting Information Available

Author contribution details; PL spectra of the supernatants before and after the hydrothermal reaction; XEOL, RT-PL, FTIR, Raman, UPS, and fitted PLE spectra of ZGO:Cr and ZGO:Cr-SiC NPs; phonon modes of zinc gallate; an extreme-condition model for quantifying growth kinetics; quantum yield of ZGO:Cr-SiC NPs as a function of excitation wavelength; STEM and HRTEM images of ZGO:Cr-SiC; details of characterization methods including XRPD, XEOL, XPS, UPS, UV–vis spectroscopy, fluorescence spectroscopy, QY measurements, X-band ESR measurements, atomic absorption spectrometry, HRTEM, SEM, DLS, and Raman and low-temperature PL measurements (PDF)

## Notes

Full description of author contribution can be found in the Supporting Information.
