# Supplementary material for: Enhancement of X-ray-Excited Red Luminescence of Chromium-Doped Zinc Gallate via Ultrasmall Silicon Carbide Nanocrystals
Source: Chem Mater. 2021 Mar 18;33(7):2457–65. doi: 10.1021/acs.chemmater.0c04671 (PMC8042637; doi:10.1021/acs.chemmater.0c04671)
Supplement: Supplementary file 1 — cm0c04671_si_001.pdf [file cm0c04671_si_001.pdf]

Supplementary Information

for the article entitled

Enhancement of X-Ray Excited Red Luminescence of Chromium Doped Zinc Gallate via Ultrasmall Silicon Carbide Nanocrystals

Dávid Beke,[a,b,*⊥] Marco V. Nardi,[c,*⊥] Gábor Bortel,[a] Melanie Timpel,[c,*⊥] Zsolt Czigány,[d] Luca Pasquali,[e,f,g] Andrea Chiappini,[h] Giorgio Bais,[I] Mátyás Rudolf,[a] Dóra Zalka,[a] Franca Bigi,[j,k] Francesca Rossi,[k] Laszlo Bencs,[a] Aron Pekker,[a] Bence G. Márkus,[a,l] Giancarlo Salviati,[k] Stephen E. Saddow,[m] Katalin Kamarás,[a] Ferenc Simon,[l] Adam Gali[a,b,*]

[a.] Institute for Solid State Physics and Optics, Wigner Research Centre for Physics, PO. Box 49, H-1525, Hungary

[b.] Department of Atomic Physics, Budapest University of Technology and Economics, Budafoki út 8., Budapest, H-1111, Hungary

[c.] IMEM-CNR, Institute of Materials for Electronic and Magnetism, Trento unit c/o Fondazione Bruno Kessler, Via Alla Cascata 56/C, Povo – 38123 Trento, Italy.

[d.] Institute for Technical Physics and Materials Science, Centre for Energy Research, Konkoly-Thege M. út 29-33., H-1121 Budapest, Hungary

[e.] IOM-CNR Institute, Area Science Park, SS 14 Km, 163.5 – 34149 Basovizza, Trieste (Italy)

[f.] University of Modena e Reggio Emilia, Engineering Department, "E. Ferrari", Via Vivarelli 10 – 41125 Modena (Italy)

[g.] Department of Physics, University of Johannesburg, PO Box 524, Auckland Park, 2006 (South Africa)

[h.] CNR-IFN, CSMFO Lab, & FBK Photonics Unit, Via Alla Cascata 56/C, Povo – 38123 Trento (Italy)

[i.] Elettra - Sincrotrone Trieste, S.C.p.A., Area Science Park, Basovizza, SS 14 Km 163.5 – 34149, Trieste (Italy)

[j.] Dipartimento di Scienze Chimiche, della Vita e della Sostenibilità Ambientale, Università di Parma, 43124 Parma, Italy

[k.] IMEM Parma-CNR, 43124 Parma, Italy

[l.] Department of Physics, Budapest University of Technology and Economics and MTA-BME Lendület Spintronics Research Group (PROSPIN), Budafoki út 8., Budapest, H-1111, Hungary

[m.] Department of Electrical Engineering, University of South Florida, 4202 East Fowler Ave., Tampa, Florida 33620, USA

[⊥] these authors contributed equally on the manuscript.

*Corresponding authors: Dávid Beke: beke.david@wigner.hu, Melanie Timpel: melanie.timpel@imem.cnr.it, Marco V. Nardi: marcovittorio.nardi@unitn.it, Adam Gali: gali.adam@wigner.hu

We checked the presence of ultrasmall SiC NPs (1-3 nm, SiC-I) and larger SiC NPs (4-6 nm, SiC-II) in the liquid phase before and after the reaction at [SiC] of 9×10^{-6} mol/L to confirm if SiC precipitates with the hydroxides. The precipitate was centrifuged down after ammonia was added to the solution and stirred for 30 min. A separate fraction of the solution was stored in the dark for two days before centrifugation. The PL spectrum of ZGO:Cr precursor supernatant showed no emission. The PL spectra of the stored ZGO:Cr-SiC-I and ZGO:Cr-SiC-II supernatants,

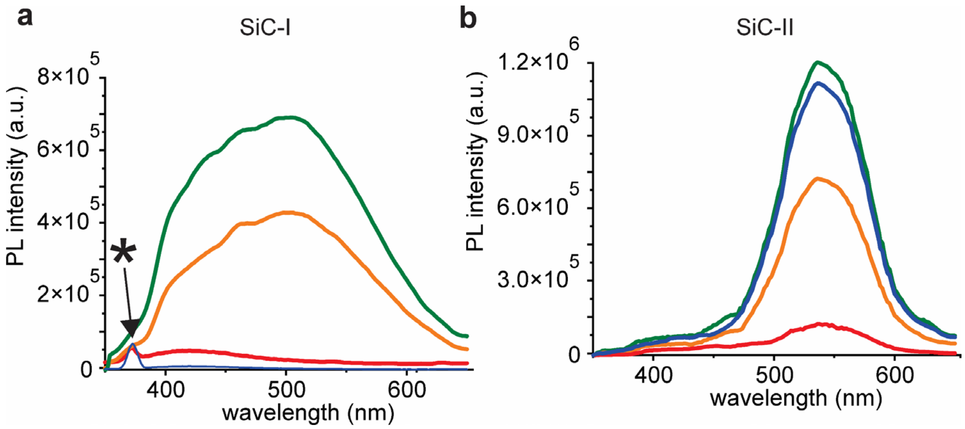

Figure S1. The PL spectra of the supernatants before and after hydrothermal reaction. **a** shows the response from 1-3 nm SiC NPs (SiC-I), while **b** is for 4-6 nm SiC NPs (SiC-II). Spectra in green represent the original SiC NP solutions. The NP concentration was adjusted to the reaction concentration. Spectra in red represent the supernatant removed from the $Zn(NO_3)_2$, $Ga(NO_3)_3$, $Cr(NO_3)_3$ SiC system immediately after the pH was adjusted to 9 and any hydroxides were precipitated. Orange lines represent the supernatants that were removed after keeping the basic solution overnight. The PL spectra of the supernatant after hydrothermal reaction are shown in blue. The water Raman peak is marked by an asterisk.

and the supernatant removed immediately after 30 min of stirring, can be seen in Figs. S1a and S1b, and compared to the PL spectra of the original SiC solutions. The supernatant removed right after stirring had a significantly lower PL intensity than either the supernatant stored overnight or the original SiC solutions indicating that SiC precipitates with the hydroxide and can be redispersed after some time. The SiC-II NPs (size 4-6 nm) do not incorporate into the ZGO:Cr NPs that is seen on the the PL intensity of the supernatant. After the reaction, the PL intensity is comparable to the original solution.

Additional XEOL and PL spectra

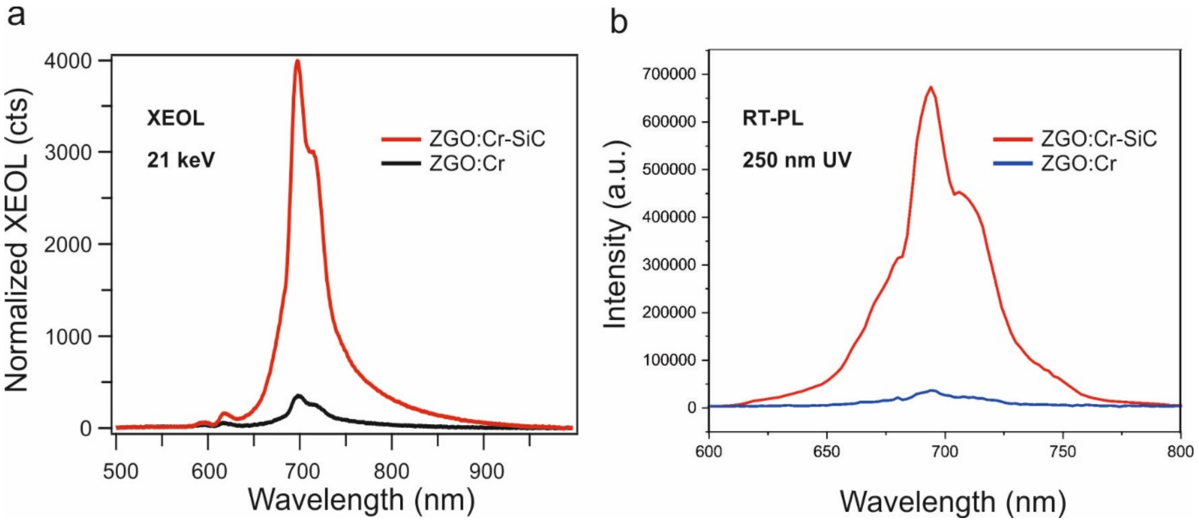

Figure S2. (a) XEOL spectra (monochromatized X-rays, 21 keV) and (b) RT-PL spectra (250 nm UV illumination) of ZGO:Cr and ZGO:Cr-SiC NPs.

Vibrational spectroscopic characteristics

The spinel structure-related phonon modes of ZGO are $\Gamma = A_{1g} + E_g + T_{1g} + 3T_{2g} + 2A_{2u} + 2E_u + 5T_{1u} + 2T_{2u}$. The infrared-active modes are the $4T_{1u}$, and the first-order Raman active modes are the $A_{1g} + E_g + 3T_{2g}$ modes. The FTIR spectra show two T_{1u} TO modes (Figure S2a) in the studied range.

The samples differed in their peak ratios of ν_1 and ν_2 (1.3 for ZGO:Cr and 1.1 for ZGO:Cr-SiC, respectively). Unfortunately, only insufficient reports analyzed the correlation between the IR active bands and the crystal structure, and they associated the decreased ratio to the increased

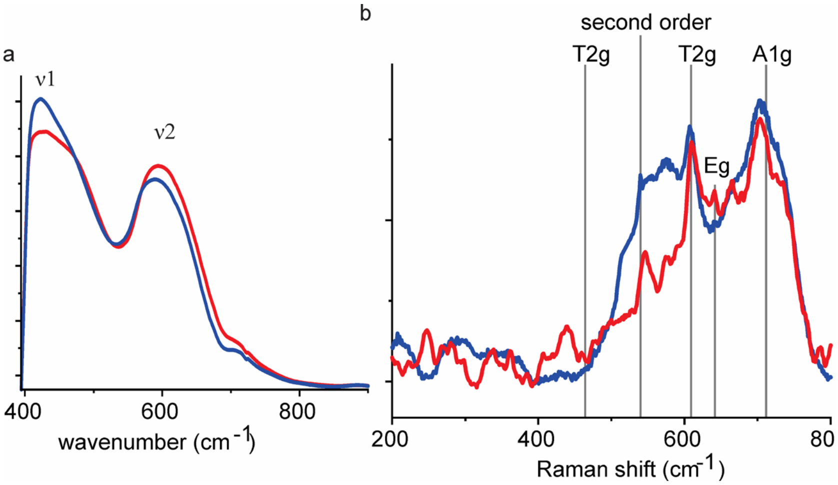

Figure S3. FTIR spectra (a) and Raman spectra (b) of ZGO:Cr and ZGO:Cr-SiC. Blue lines represent the ZGO:Cr sample and red lines are for ZGO:Cr-SiC.

inversion disorder in the crystal [1]. That is evident in the Raman spectra where, among the most prominent T_{2g} and A_{1g} related peaks, ZGO:Cr-SiC contains discernible peaks at 440 cm^{-1}, 545 cm^{-1}, 665 cm^{-1}, and 727 cm^{-1}. The peaks around 540 cm^{-1} and 670 cm^{-1} can be associated with the appearance of second-order vibrations[2,3] and indicate the presence of an inverse spinel structure. The prominence of these peaks in ZGO:Cr-SiC is similar to a previous report considering a Si-doped zinc gallate structure[4]. The detected peaks and their origin, compared to the literature data, are listed in Table S1. As can be seen, some of the additional peaks found in the Raman spectra coincide with the reported vibrational frequencies determined only from the optical spectrum of Cr^{3+}. The presence of forbidden transitions in ZGO:Cr indicates lower crystallinity.

Table S1. Phonon modes of zinc gallate. Literature data are from Refs. [2,3,5,6].

Point group symmetry	Detected peaks/cm^{-1} (literature)	Detection method	ZGC	ZGC-SiC
	140	PL		
T_{1u}	175-180	FTIR<br>PL	-	

	258	PL	-	
T_{1u}	325-335	FTIR<br>PL	-	
T_{1u}	418-455	FTIR<br>PL	422 FTIR	430 FTIR<br>440 Raman
Second-order	467	Raman		
Second-order	532	Raman<br>PL		545 Raman
T_{1u}	570-593	FTIR<br>PL	577 Raman<br>591 FTIR	595 FTIR
T_{2g}	611	Raman	607 Raman	611 Raman
E_g	638	Raman		640 Raman
	680	PL		660 Raman
A_{1g}	714	Raman	705 Raman	705 Raman
			730 Raman	730 Raman

Energy levels in ZGO:Cr -SiC

Depending on the excitation energy, one can excite electrons from the ground states to one of the excited states. Such excitations are usually present in the PLE spectra, and the relative positions between the ground and excited states can be calculated. The PLE contains all the allowed transitions: $^4A_2 \rightarrow {}^4T_1$, $^4T_2$, CB (ZGO:Cr), CB (SiC) (if possible). UPS gives information about the position of the Fermi level, relative to the valence band maximum, and the absolute position of the valence band maximum of the system. The Fermi level has to be below the excited states and above the ground state.

The bandgap of SiC NPs has been determined from their PLE and PL spectra[7]. SiC NPs have a size- and surface-dependent optical bandgap that can vary between 3.1-3.9 eV for ultrasmall SiC NPs (< 3nm). However, the excitonic emission bandgap varied between 2.75-3.1 eV due to surface states. The ZGO:Cr-SiC NPs displayed a 430 nm emission maximum upon 330-nm excitation, which implies an optical bandgap of 2.9 eV and an excitonic band gap of 3.7 eV, respectively. Such values match with the bandgap of SiC NPs that are coordinated with metallic ions[7,8] suggesting similar interface states. To estimate the energy level positions shown in Fig. 6 of the main text, we used the optical bandgap for the SiC core and the excitonic bandgap represented by its interface states. The bandgap value of ZGO:Cr was obtained from literature reports[9–12]. Despite the uncertainty of the bandgap determination for SiC and ZGO:Cr NPs, the present PLE, PL, and UPS data, when taken together, can determine the absolute positions of the electronic levels.

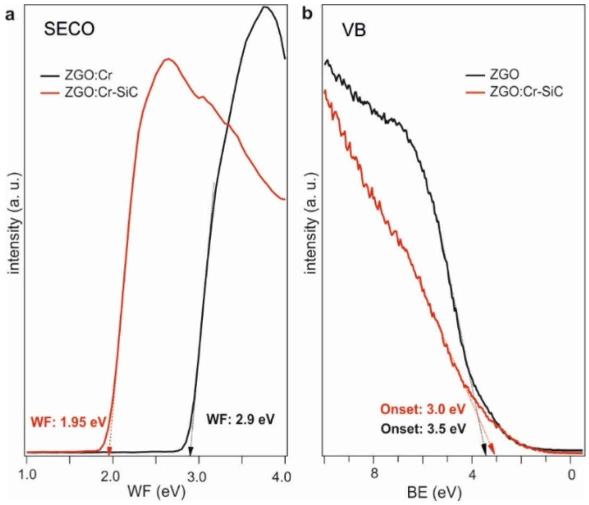

Figure S4. a Secondary electron cutoff (SECO) and **b** valence band (VB) region UPS spectra of ZGO:Cr and ZGO:Cr-SiC NPs. The WF and BE onsets are determined from x-axis intercepts as indicated.

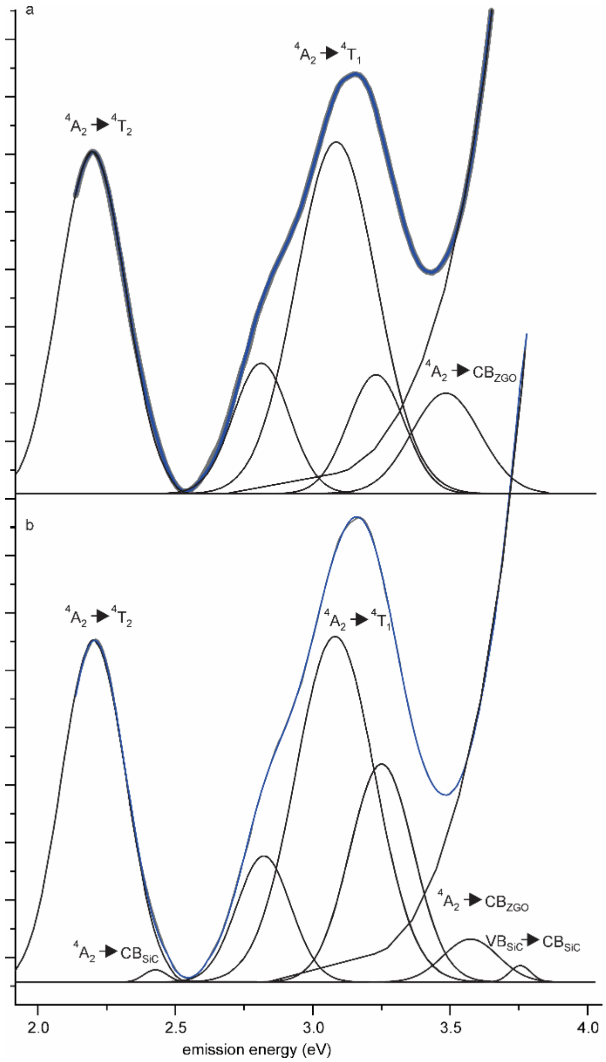

Figure S5. Fitted PLE spectra of **a** ZGO:Cr and **b** ZGO:Cr-SiC.

Extreme-condition model for quantifying growth kinetics

The growth kinetics parameters (k and D) were not calculated for ZGO:Cr and ZGO:Cr-SiC NP formation as the sampling method was necessary for the long reaction time used and did not provide enough data points in the critical regions for accurate calculation. The difference between

the reaction kinetics for early reaction conditions is easy to observe through the growth rate. According to the model, the slope of the $\ln\left(V_{\text{tot,max}}^{1/3} - V_{\max}^{1/3}\right) \sim t$ plot at the very late stage of growth is proportional to D meaning that comparing the slope at the late growth stage gives information about a difference in the reaction conditions. Fig. S6 shows the $\ln\left(V_{\text{tot,max}}^{1/3} - V_{\max}^{1/3}\right) - t$ plot and it can be seen that there is only a marginal difference in the slopes of ZGO:Cr and ZGO:Cr-SiC NP formation thus proposing similar reaction kinetics, i.e., the same diffusion constant at the diffusion limited stage.

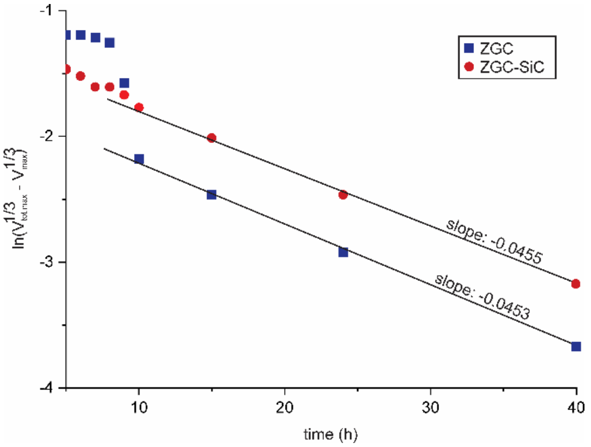

Figure S6. Plot for calculating the reaction condition at the very late stage of the reaction.

Quantum yield of ZGO:Cr -SiC NPs as function of excitation wavelength

The quantum yield (QY) of ZGO:Cr-SiC NPs at a wavelength of 250 nm is almost twice as large as that at wavelength at 290 nm as shown in Fig. S7.

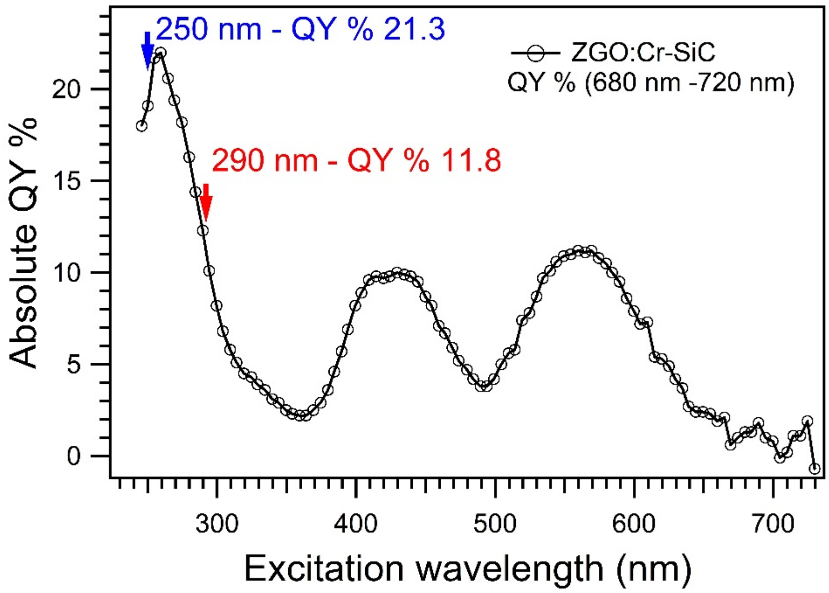

Figure S7. Quantum yield (QY) of ZGO:Cr-SiC NPs as function of excitation wavelength (integration interval: 680 – 720 nm). The wavelengths used for UV illumination of the ZGO:Cr and ZGO:Cr-SiC NPs are marked by arrows.

Identification of the core-shell structure

Identifying the core-shell structure was primarily based on HR-TEM images, where lighter elements, such as Si and C, compared to heavier Zn and Ga, created a brighter middle zone in the image if the particle had a SiC core (or a darker middle zone in the image in high angle angular dark-field (HAADF) mode). Due to the rapid nucleation process, seed-free nucleation of ZGO/ZGO:Cr can occur even when the SiC concentration is high enough for seeded-only nucleation (see Figure 5a). We sorted the particles from several TEM images, and observed about 80% seeded nucleation in the ZGO:Cr-SiC system and less than 2 % for the ZGO:Cr system (where probably some pollutant particles acted as a seed). The core size varied between 2 to 4 nm, correlating well with the size of the SiC NPs (1 - 3 nm). We did not observe any indication of bare SiC NPs or that SiC NPs had absorbed onto the surface of the ZGO NPs.

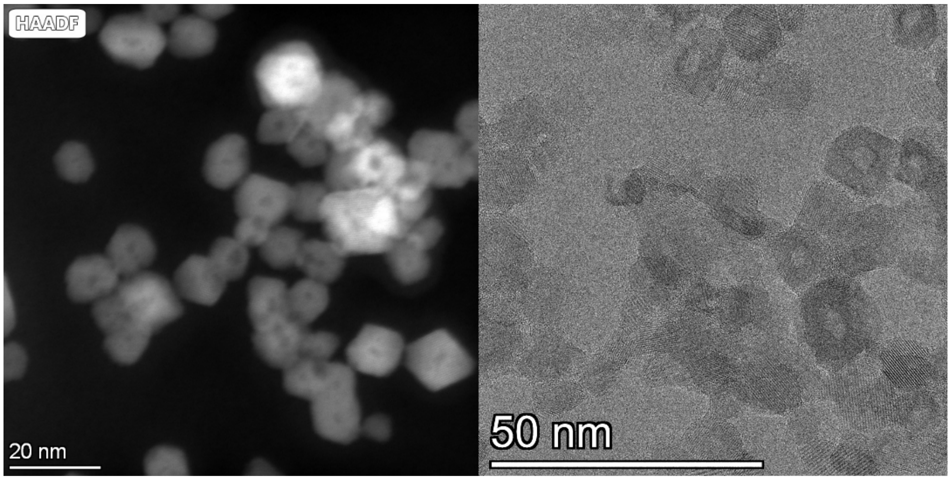

Figure S8. High angle angular dark field (STEM) and HR-TEM images of ZGO:Cr-SiC.

Our reaction-kinetics study also confirmed the seeded nucleation results in a core-shell system (see Figure 5b-e in the main text). We also followed the reaction with Raman spectroscopy, and the Raman spectra clearly showed the development of ZGO related peaks along with a decrease in SiC related peaks.

As a reference, we mixed the same amount of SiC and ZGO:Cr NPs, and we measured the PL of the colloid solution and of a pellet made from the same mixture. Afterwards we measured the XEOL of the pellet. We did not observe the reported sensitizing effect when the two systems were mixed after the reaction.

Methods

X-ray powder diffraction (XRPD) data were measured on a Huber G670 Guinier Imaging Plate Camera using Cu Kα_1 radiation (λ = 1.54056 Å) from a focusing Johannson monochromator. During the measurements the samples were held in glass capillaries of 0.5 mm diameter and 0.01 mm wall thickness at room temperature. The Rietveld refinements were performed using the program Topas Academic (by Alan A. Coleho http://www.topas-academic.net/).

X-ray excited optical luminescence (XEOL) measurements with non-monochromatized X-ray radiation were performed using a conventional sealed tube X-ray source and an Ocean Optics QE Pro-FL spectrometer (Wavelength range: 350 – 1100 nm, Optical resolution (HC1 grating, 200 μm slit): ~6.4 nm. The unfiltered, non-monochromatized X-ray radiation from the X-ray source contained both the characteristic radiation of the Cr anode (Cr Kα at 5.4 keV, Cr Kβ at 5.9 keV), and continuous bremsstrahlung radiation up to 40 keV energy. The Be window of the source and the 5 cm air-path from the source to the sample effectively absorbed the radiation below 3 keV photon energy. The sample was placed on a flat substrate and was irradiated with spot size of about 1 cm. The emitted light was collected by a QP600-025-UV-BX optical fiber. Typical measurements took 10-60 seconds.

XEOL measurements with monochromatized high-flux X-ray radiation were performed at the XRD1 beamline of the ELETTRA synchrotron facility in Trieste (Italy) using an excitation energy of 21.0 keV and a flux of 6.4×10^{11} photon/s. A high Sensitivity Fluorescence Spectrometer QEPRO-FL (Ocean Insight) equipped with a back-thinned, thermoelectrically cooled CCD array detector (entrance slit: 200 μm; optical resolution: 6.87 nm FWHM; wavelength range: 350 nm - 1.1 μm) coupled with a solarization-resistant Ocean Insight optical fibre (core size: 1000 μm) was used to collect the XEOL signal.

X-ray photoelectron spectroscopy (XPS) was performed with a non-monochromatized Mg Kα source (emission line at 1253.6 eV), and **ultraviolet photoelectron spectroscopy** (UPS) was carried out using the He I photon with an energy of 21.2 eV. The photoelectron signals were detected with a VSW HSA100 hemispherical analyzer equipped with a PSP electronic power supply and control. The total energy resolution was about 0.8 eV for XPS and 0.1 eV for UPS. The binding energy (BE) scale of the XPS spectra was calibrated using the Au 4f peak at 84.0 eV as a

reference, whereas UPS BEs were referred to the Fermi level of the same clean Au substrate. The secondary electron cutoff (SECO) spectra were measured with a sample bias of −7.0 eV.

UV-VIS spectroscopy (Ocean Optics, DH-2000-BAL light source, and QE5000 spectrometer) was used to check the absorbance of the aqueous colloidal solutions. **Fluorescence spectroscopy** (PL) and photoluminescence excitation spectroscopy (PLE) measurements were performed on a Horiba Jobin-Yvon NanoLog FL3-2iHR spectrophotometer equipped with 450 W Xenon lamp, iHR-320 grating spectrometer, and a R928P photomultiplier tube. In the case of colloids, the measurements were carried out in a 10 mm quartz cuvette (Hellma 110-QS). Dry samples were prepared by making 1.1 cm diameter pellets by pressing the powder with a hydraulic press at 3 tons. The PLE was recorded at 695 nm emission wavelength at a slit size of 10 nm.

For the 250 nm UV illumination, we used a DH-200-BAL deuterium light source and a 400 nm short-pass filter (Thorlabs FES400) and the iHR-320 grating spectrometer along with the R928P photomultiplier tube of the Horiba instrument. The shortest wavelength in the Horiba instrument is 260 nm. The deuterium lamp has an intensity maximum at 250 nm.

Quantum Yield (QY) measurements were obtained using a Hamamatsu Quantaurus-QY C11347-11, equipped with a xenon lamp with a nominal power of 150 W. Considering the emission of Cr in the range between 600 and 800 nm, different excitation wavelengths, in the range 250-750 nm, were employed. The QY values were determined by measuring the ratio between the number of photons emitted from the sample (PN_{em}) and those absorbed by the sample (PN_{abs}).

We studied **X-band ESR** (0.35 T, 9.4 GHz) in a commercial spectrometer (Bruker Elexsys E500). Samples were placed in high-quality, defect-free quartz ampoules. Special care was taken to employ a low microwave excitation power (0.2 mW) with low magnetic field (0.15-0.25 G)

modulation to avoid any distortion to the ESR lineshapes. The measurements were carried out in the dark at room temperature, so the charge state of the defects was determined in thermal equilibrium ambient conditions.

The Cr content of the doped ZGO and SiC NPs suspended in aqueous solutions was quantitated by means of **high resolution continuum source graphite furnace atomic absorption spectrometry** (HR-CS-GFAAS). To this end, an interference-free method was optimized for Cr determination, including the spectral conditions (analytical wavelength: Cr I 357.8687 nm, spectral resolution: 6.0 pm at 3-pixel CCD evaluation) and the graphite furnace heating program. For the latter, a three-step drying stage spanning from 80 to 110 °C with pyrolysis and atomization temperatures of 350 °C, 1000 °C, and 2400 °C, respectively, were applied, each with optimized heating and holding time. A 20 μL aliquot of each sample solution was directly injected into the graphite atomizer by the assistance of an MPE-60 autosampler. Before this step, each sample solution was shortly shaken in order to attain homogeneous nano-particle dispersion, thus a repeatable, representative sample introduction for the AAS-method was used. Due to the relatively high Cr dopant content, the sample solutions were analyzed after 10- or 100-fold dilution with high purity (18 MΩ cm) MilliQ water. The calibration was performed against seven standard solutions, each diluted freshly from an ICP multi-element standard solution IV (Merck) using MilliQ water, after slight acidification with 0.2% Merck Suprapur HNO_3. Blank solutions were prepared from the reagents and from nano-suspensions of pure (undoped) crystal samples. Triplicate measurement cycles were performed for each sample and standard, from which the average and the standard deviation values were calculated. Rectilinear calibration curves were obtained up to 20 μg/L Cr with regression coefficients of at least 0.9995. The method limit of

detection was 0.28 μg/L. The precision of the determinations – expressed as the relative standard deviation – was not worse than 5.8% and 4.2% for the sample and standard solutions, respectively.

High resolution transmission electron microscopy (HRTEM). The nanoparticles were drop dried on TEM grids covered by a 3-nm thick carbon film (Ted Pella 01824). The HRTEM investigations were performed in a C_S corrected Themis (Thermo Fischer; USA) instrument operated at 200 kV and point resolution of 0.8 Å.

Scanning electron microscopy images and electronic dispersive spectroscopic data was recorded by a TESCAN MIRA3 electron microscope.

Dynamic light scattering (DLS, Malvern Nano S) was used for size distribution characterization. The aqueous solution was used without the addition of surfactant and concentration optimization.

Raman and low temperature PL measurements were carried out with a Renishaw InVia confocal Raman microscope and a 2 W continuous 532-nm laser source at 10% intensity.

References

(1) Basak, D.; Ghose, J. Infrared Studies on Some Substituted Copper Chromite Spinels. *Spectrochim. Acta Part A Mol. Spectrosc.* **1994**, *50* (4), 713–718. https://doi.org/10.1016/0584-8539(94)80008-1.

(2) Can, M. M.; Hassnain Jaffari, G.; Aksoy, S.; Shah, S. I.; Firat, T. Synthesis and Characterization of ZnGa2O4 Particles Prepared by Solid State Reaction. *J. Alloys Compd.* **2013**, *549*, 303–307. https://doi.org/10.1016/j.jallcom.2012.08.137.

(3) Wani, S.; Sofi, H. S.; Sheikh, F. A.; Shivashankar, S. A.; Majeed, S. ZnGa2O4 Nanophosphors: Rapid Synthesis, Characterization and Luminescence Properties. *Mater. Sci. Res. India* **2017**, *14* (2), 116–122. https://doi.org/10.13005/msri/140205.

(4) Kang, H. I.; Kim, J. S.; Lee, M.; Bahng, J. H.; Choi, J. C.; Park, H. L.; Kim, G. C.; Kim, T. W.; Hwang, Y. H.; Mho, S. I.; et al. Tunable Color Emission of ZnGa2O4:Si4+ Phosphors with Enhanced Brightness Due to Donor Formation. *Solid State Commun.* **2002**, *122* (12), 633–636. https://doi.org/10.1016/S0038-1098(02)00235-1.

(5) Kahan, H. M.; Macfarlane, R. M. Optical and Microwave Spectra of Cr 3+ in the Spinel ZnGa 2 O 4. *J. Chem. Phys.* **1971**, *54* (12), 5197–5205. https://doi.org/10.1063/1.1674815.

(6) Ramesh Kumar, V.; Narasimhulu, K. V.; Gopal, N. O.; Jung, H. K.; Chakradhar, R. P. S.;

Rao, J. L. EPR, Luminescence and IR Studies of Mn Activated ZnGa2O 4 Phosphor. *J. Phys. Chem. Solids* **2004**, *65* (7), 1367–1372. https://doi.org/10.1016/j.jpcs.2004.03.009.
(7) Beke, D.; Fučíková, A.; Jánosi, T. Z.; Károlyházy, G.; Somogyi, B.; Lenk, S.; Krafcsik, O.; Czigány, Z.; Erostyák, J.; Kamarás, K.; et al. Direct Observation of Transition from Solid-State to Molecular-Like Optical Properties in Ultrasmall Silicon Carbide Nanoparticles. *J. Phys. Chem. C* **2018**, *122* (46), 26713–26721. https://doi.org/10.1021/acs.jpcc.8b07826.
(8) Beke, D.; Jánosi, T. Z.; Somogyi, B.; Major, D. Á.; Szekrényes, Z.; Erostyák, J.; Kamarás, K.; Gali, A. Identification of Luminescence Centers in Molecular-Sized Silicon Carbide Nanocrystals. *J. Phys. Chem. C* **2016**, *120* (1), 685–691. https://doi.org/10.1021/acs.jpcc.5b09503.
(9) Cha, J. H.; Choi, H. W. Luminescence Characteristics of ZnGa2O4:Mn2+,Cr3+ Phosphor and Thick Film. *Trans. Electr. Electron. Mater.* **2011**, *12* (1), 11–15. https://doi.org/10.4313/TEEM.2011.12.1.11.
(10) Gu, Z.; Liu, F.; Li, X.; Howe, J.; Xu, J.; Zhao, Y.; Pan, Z. Red, Green, and Blue Luminescence from ZnGa2O4 Nanowire Arrays. *J. Phys. Chem. Lett.* **2010**, *1* (1), 354–357. https://doi.org/10.1021/jz900213p.
(11) Li, L.; Pan, F.; Tanner, P. A.; Wong, K. L. Tunable Dual Visible and Near-Infrared Persistent Luminescence in Doped Zinc Gallogermanate Nanoparticles for Simultaneous Photosensitization and Bioimaging. *ACS Appl. Nano Mater.* **2020**, *3* (2), 1961–1971. https://doi.org/10.1021/acsanm.9b02613.
(12) Tsai, S. H.; Basu, S.; Huang, C. Y.; Hsu, L. C.; Lin, Y. G.; Horng, R. H. Deep-Ultraviolet Photodetectors Based on Epitaxial ZnGa2O4 Thin Films. *Sci. Rep.* **2018**, *8* (1), 1–9. https://doi.org/10.1038/s41598-018-32412-3.

Author contributions

Manuscript title: Enhancement of X-Ray Excited Red Luminescence of Chromium Doped Zinc Gallate via Ultrasmall Silicon Carbide Nanocrystals

All authors discussed the results and contributed to the final manuscript

Author 1: Dávid Beke

☒ Conceived and designed the analysis
Specify contribution in more detail (optional; no more than one sentence)

☒ Collected the data
Specify contribution in more detail (optional; no more than one sentence)

☒ Contributed data or analysis tools
PL, DLS, XEOL, Raman, FTIR

☒ Performed the analysis
Contributed in PL, DLS, XEOL, Raman, FTIR, TEM, ESR, kinetic study analysis

☒ Wrote the paper
Wrote the initial draft of the manuscript and coordinated the revision.

☒ Other contribution
Prepared and visualized the data for publication.

Author 2: Marco V. Nardi

☐ Conceived and designed the analysis
Specify contribution in more detail (optional; no more than one sentence)

☒ Collected the data
Performed the XEOL, QY, XPS, and UPS measurements.

☒ Contributed data or analysis tools
Provided access to XEOL, XPS, and UPS instruments.

☒ Performed the analysis
Analysed the XEOL, QY, XPS, and UPS data.

☒ Wrote the paper
Critically reviewed the manuscript.

☒ Other contribution
Prepared and visualized the data for publication.

Author 3: Gábor Bortel

☐ Conceived and designed the analysis
Specify contribution in more detail (optional; no more than one sentence)

☐ Collected the data
Provided access to the XRD instruments

☒ Contributed data or analysis tools
Performed the XPS measurement and contributed to the XEOL measurements (Elettra beamline)

☒ Performed the analysis
Analysed the XPS data

☐ Wrote the paper
Critically reviewed the manuscript.

☐ Other contribution
Specify contribution in more detail (required; no more than one sentence)

Author 4: Melanie Timpel

☐ Conceived and designed the analysis
Specify contribution in more detail (optional; no more than one sentence)

☐ Collected the data
Specify contribution in more detail (optional; no more than one sentence)

☒ Contributed data or analysis tools
Assisted in XEOL, XPS, and UPS data acquisition.

☐ Performed the analysis

☒ Wrote the paper
Participated in manuscript writing, critically reviewed the manuscript.

☒ Other contribution
Prepared and visualized the data for publication.

Author 5: Zsolt Czigány

☐ Conceived and designed the analysis
Specify contribution in more detail (optional; no more than one sentence)

☐ Collected the data
Specify contribution in more detail (optional; no more than one sentence)

☒ Contributed data or analysis tools
Provided access to TEM and performed the TEM and EDS measurements

☒ Performed the analysis
Analysed the TEM and EDS data

☐ Wrote the paper
Specify contribution in more detail (optional; no more than one sentence)

☐ Other contribution
Specify contribution in more detail (required; no more than one sentence)

Author 6: Luca Pasquali

☐ Conceived and designed the analysis
Specify contribution in more detail (optional; no more than one sentence)

☒ Collected the data
performed the UPS and XEOL (Elettra beamline) measurements

☒ Contributed data or analysis tools
Granted access to the detector for XEOL measurements at the Elettra beamline.

☒ Performed the analysis
Analyzed the UPS and XEOL (Elettra beamline) data.

☐ Wrote the paper
Specify contribution in more detail (optional; no more than one sentence)

☐ Other contribution
Specify contribution in more detail (required; no more than one sentence)

Author 7: Andrea Chiappini

☐ Conceived and designed the analysis
Specify contribution in more detail (optional; no more than one sentence)

☒ Collected the data
Conducted the acquisition of QY data.

☒ Contributed data or analysis tools
Supervised the instrument for QY analysis.

☒ Performed the analysis
QY

☐ Wrote the paper
Specify contribution in more detail (optional; no more than one sentence)

☐ Other contribution
Specify contribution in more detail (required; no more than one sentence)

Author 8: Giorgio Bais

- ☐ Conceived and designed the analysis
  Specify contribution in more detail (optional; no more than one sentence)
- ☐ Collected the data
  Specify contribution in more detail (optional; no more than one sentence)
- ☒ Contributed data or analysis tools
  Granted access to Elettra beamline for XEOL acquisition and supervised the beamline and the measurements.
- ☐ Performed the analysis
  Specify contribution in more detail (optional; no more than one sentence)
- ☐ Wrote the paper
  Specify contribution in more detail (optional; no more than one sentence)
- ☐ Other contribution
  Specify contribution in more detail (required; no more than one sentence)

Author 9: Mátyás Rudolf

- ☐ Conceived and designed the analysis
  Specify contribution in more detail (optional; no more than one sentence)
- ☐ Collected the data
  Specify contribution in more detail (optional; no more than one sentence)
- ☒ Contributed data or analysis tools
  Participated in PL, Raman, and XEOL measurements, nanoparticle synthesis
- ☒ Performed the analysis
  Participated in PL and XEOL data analysis.
- ☐ Wrote the paper
  Specify contribution in more detail (optional; no more than one sentence)
- ☐ Other contribution
  Specify contribution in more detail (required; no more than one sentence)

Author 10: Dóra Zalka

☐ Conceived and designed the analysis
Specify contribution in more detail (optional; no more than one sentence)

☐ Collected the data
Specify contribution in more detail (optional; no more than one sentence)

☒ Contributed data or analysis tools
Provided access to the SEM and performed the SEM, EDS measurements

☒ Performed the analysis
Analysed SEM, EDS data and participated in the DLS data analysis

☐ Wrote the paper
Specify contribution in more detail (optional; no more than one sentence)

☐ Other contribution
Specify contribution in more detail (required; no more than one sentence)

Author 11: Franca Bigi

☐ Conceived and designed the analysis
Specify contribution in more detail (optional; no more than one sentence)

☐ Collected the data
Specify contribution in more detail (optional; no more than one sentence)

☒ Contributed data or analysis tools
Contributed to Nanoparticle synthesis

☒ Performed the analysis
Colloid stability, reaction kinetic

☐ Wrote the paper
Specify contribution in more detail (optional; no more than one sentence)

☐ Other contribution
Contributed to the synthesis design

Author 12: Francesca Rossi

☐ Conceived and designed the analysis
Specify contribution in more detail (optional; no more than one sentence)

☐ Collected the data
Specify contribution in more detail (optional; no more than one sentence)

☒ Contributed data or analysis tools
Reviewed the TEM, EDS measurements

☒ Performed the analysis
Contributed to TEM, SEM, EDS, data analysis

☐ Wrote the paper
Specify contribution in more detail (optional; no more than one sentence)

☐ Other contribution
Specify contribution in more detail (required; no more than one sentence)

Author 13: László Bencs

☐ Conceived and designed the analysis
Specify contribution in more detail (optional; no more than one sentence)

☒ Collected the data
Performed the AAS measurements

☒ Contributed data or analysis tools
Provided access AAS device

☒ Performed the analysis
Developed the AAS method for the NPs and analysed the AAS data

☐ Wrote the paper
Specify contribution in more detail (optional; no more than one sentence)

☐ Other contribution
Specify contribution in more detail (required; no more than one sentence)

Author 14: Aron Pekker

☐ Conceived and designed the analysis
Specify contribution in more detail (optional; no more than one sentence)

☒ Collected the data
Raman, LTPL

☒ Contributed data or analysis tools
Provided access to Raman and the cryostat,

☒ Performed the analysis
Contributed to the Raman and FTIR analysis

☐ Wrote the paper
Specify contribution in more detail (optional; no more than one sentence)

☐ Other contribution
Specify contribution in more detail (required; no more than one sentence)

Author 15: Bence G. Márkus

☐ Conceived and designed the analysis
Specify contribution in more detail (optional; no more than one sentence)

☒ Collected the data
Performed the ESR measurements

☐ Contributed data or analysis tools

☒ Performed the analysis
Contributed to ESR analysis

☐ Wrote the paper
Specify contribution in more detail (optional; no more than one sentence)

☐ Other contribution
Specify contribution in more detail (required; no more than one sentence)

Author 16: Giancarlo Salviati

☐ Conceived and designed the analysis
Specify contribution in more detail (optional; no more than one sentence)

☐ Collected the data
Specify contribution in more detail (optional; no more than one sentence)

☐ Contributed data or analysis tools

☒ Performed the analysis
Contributed to XEOL analysis and experiment designs

☐ Wrote the paper
Specify contribution in more detail (optional; no more than one sentence)

☒ Other contribution
Financially supported the experiments by providing materials and reagents.

Author 17: Stephen E. Saddow

☒ Conceived and designed the analysis
Contributed to the design of the experiments

☐ Collected the data
Performed the XEOL measurements

☐ Contributed data or analysis tools

☐ Performed the analysis

☐ Wrote the paper
Critically reviewed the paper.

☒ Other contribution
Supervised the research, supported the entire project.

Author 18: Katalin Kamarás

☐ Conceived and designed the analysis
Specify contribution in more detail (optional; no more than one sentence)

☐ Collected the data
Specify contribution in more detail (optional; no more than one sentence)

☐ Contributed data or analysis tools
Provided access to the FTIR, PL, and Raman instruments,

☒ Performed the analysis
Supervised the Raman and FTIR analysis

☒ Wrote the paper
Specify contribution in more detail (optional; no more than one sentence)

☒ Other contribution
Critically reviewed the theoretical part of the paper and the results

Author 19: Ferenc Simon

☐ Conceived and designed the analysis
Specify contribution in more detail (optional; no more than one sentence)

☐ Collected the data
Specify contribution in more detail (optional; no more than one sentence)

☒ Contributed data or analysis tools
Provided access to ESR instrument

☒ Performed the analysis
Contributed to the ESR analysis

☐ Wrote the paper
Specify contribution in more detail (optional; no more than one sentence)

☒ Other contribution
Supervised the ESR measurements and analysis.

Author 20: Adam Gali

☒ Conceived and designed the analysis
Specify contribution in more detail (optional; no more than one sentence)

☐ Collected the data
Specify contribution in more detail (optional; no more than one sentence)

☐ Contributed data or analysis tools

☒ Performed the analysis
Critically reviewed the final results

☒ Wrote the paper
Critically reviewed the paper.

☒ Other contribution
Supervised the project and acquired the financial support for the project leading to this publication.
